# The Equipment of Research Laboratories

**Published:** 1916-07-22

**Authors:** 


### THE EQUIPMENT OF RESEARCH LABORATORIES.

### Some Applications of Gas Heating.

Wherever large hospital laboratories are equipped for special research work, heating and water-heating facilities are very important. In the animal rooms it is necessary to provide heat practically all the year round; when the ordinary coke or coal boilers employed all over the hospitals in winter are out of use, the radiators may be equally well supplied by means of gas-boilers, which provide the hot water required for keeping the rooms at an even and steady temperature. It is claimed that by gas-boilers a much more reliable temperature is maintained. Gas-boilers for this purpose may be fixed in a special room; but as they generally occupy little space, this is not always necessary. For the provision of hot water at the sink a separate gas-heater is usually installed in addition. All these boilers can be provided with automatic governors to maintain exactly the desired temperature. Flue-pipes are, of course, necessary, and the rooms should be also adequately ventilated.

In many laboratories a good supply of gas is carried round the walls just below the working benches. At various points on these branch pipes should be taken through about 6 inches above the bench level, and two- or three-way gas-taps fitted; these are best in some oxidised or bronzed metal. From the taps the gas is supplied by rubber connections to various appliances, for glass-blowing, for evaporating liquids, for boiling, incubating, fusing metal, etc.

In the fume cupboards of chemical laboratories gas is constantly required. The cupboards are glass fronted and built on a stone bench against a wall, in which there should be a flue not less than 9 inches by 9 inches. The gas is brought to the cupboard under the bench with a branch through actually into the cupboard, and the flame of each heating burner is controlled with a valve outside it. Generally the tap is placed on the front just underneath the bench. In the cupboard the gas is used in various forms of rings and burners for dealing with the experiments conducted therein.

Many research laboratories are equipped with a permanent gas incubating chamber (in addition to small portable incubators) about 8 feet by 6 feet and 10 to 11 feet high. Such chambers are lined with tiles, shelves are fitted to the walls, and the incubator is placed in the centre of the floor. The incubator consists of a circular column, varied in size as required; it has a centre stem and the column is subdivided into a large number of surfaces for radiation purposes. A good supply of gas is brought in at the floor level, connected to the incubator and consumed by a series of jets in the centre stem; this supply is controlled by one or more valves. The chamber should be built with hollow walls and no windows; fresh air should be brought in at floor level and two flue outlets made near the ceiling. This secures the steady and constant temperature necessary for generating purposes in many research experiments.

The ice-making machine in the refrigerating chamber is often conveniently worked by a small gas-engine, which can also be employed for other power purposes, as for lathes. A gas-destructor for dealing with contaminated rubbish is also valuable for research work, and should be worked where possible in the open air.

